# Reconstruction of Iberian ceramic potteries using generative adversarial networks

**DOI:** 10.1038/s41598-022-14910-7

**Published:** 2022-06-23

**Authors:** Pablo Navarro, Celia Cintas, Manuel Lucena, José Manuel Fuertes, Rafael Segura, Claudio Delrieux, Rolando González-José

**Affiliations:** 1 Instituto Patagónico de Ciencias Sociales y Humanas, Centro Nacional Patagónico, CONICET, Bv. Almirante Brown 2915, 9120 Puerto Madryn, PC Argentina; 2 Departamento de Informática (DIT), Facultad de Ingeniería, Universidad Nacional de La Patagonia San Juan Bosco, Mitre 665, 9100 Trelew Chubut, PC Argentina; 3 IBM Research Africa, Catholic University of Eastern Africa Campus, Bogani E Rd, Nairobi, 00200 PC Kenya; 4 Department of Computer Science, University of Jaén, Campus Las Lagunillas s/n, 23071 Jaén, PC Spain; 5 Research University Institute for Iberian Archaeology, University of Jaén, Campus Las Lagunillas s/n, 23071 Jaén, PC Spain; 6 Departamento de Ingeniería Eléctricarica y de Computadoras, Universidad Nacional del Sur, and CONICET, San Andre´s 800, Campus Palihue, 8000 Bahía Blanca, PC Argentina

**Keywords:** Archaeology, Computer science

## Abstract

Several aspects of past culture, including historical trends, are inferred from time-based patterns observed in archaeological artifacts belonging to different periods. The presence and variation of these objects provides important clues about the Neolithic revolution and given their relative abundance in most archaeological sites, ceramic potteries are significantly helpful in this purpose. Nonetheless, most available pottery is fragmented, leading to missing morphological information. Currently, the reassembly of fragmented objects from a collection of thousands of mixed fragments is a daunting and time-consuming task done almost exclusively by hand, which requires the physical manipulation of the fragments. To overcome the challenges of manual reconstruction and improve the quality of reconstructed samples, we present IberianGAN, a customized Generative Adversarial Network (GAN) tested on an extensive database with complete and fragmented references. We trained the model with 1072 samples corresponding to Iberian wheel-made pottery profiles belonging to archaeological sites located in the upper valley of the Guadalquivir River (Spain). Furthermore, we provide quantitative and qualitative assessments to measure the quality of the reconstructed samples, along with domain expert evaluation with archaeologists. The resulting framework is a possible way to facilitate pottery reconstruction from partial fragments of an original piece.

## Introduction

**Figure 1:**
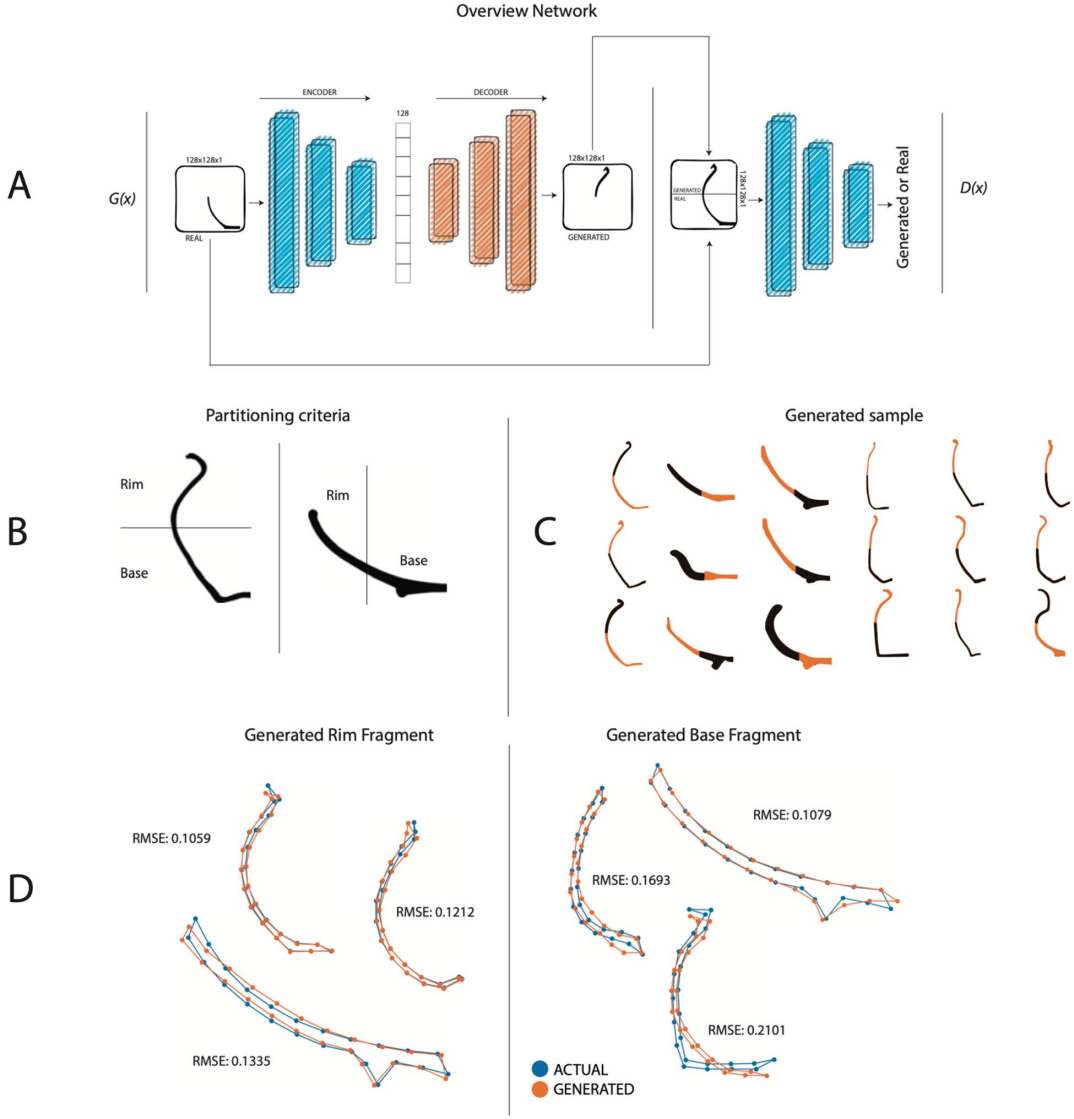
Overview of the proposed approach. (**A**) IberianGAN architecture. The *G*(*x*) generator is based on an encoder-decoder architecture. Upon receiving a fragment of pottery, the encoder transforms it into a vector and then the decoder generates the missing or unknown fragment. The discriminator *D*(*x*) receives the complete profile to determine if it is true or false. (**B**) Criteria for profile partitioning into rim and base of profiles. (**C**) Examples of IberianGAN generated samples from fragments for both open and closed shapes (shown in lighter color). (**D**) Semi-landmark analysis and RMSE values as comparing actual and artificially generated samples.

**Figure 2:**
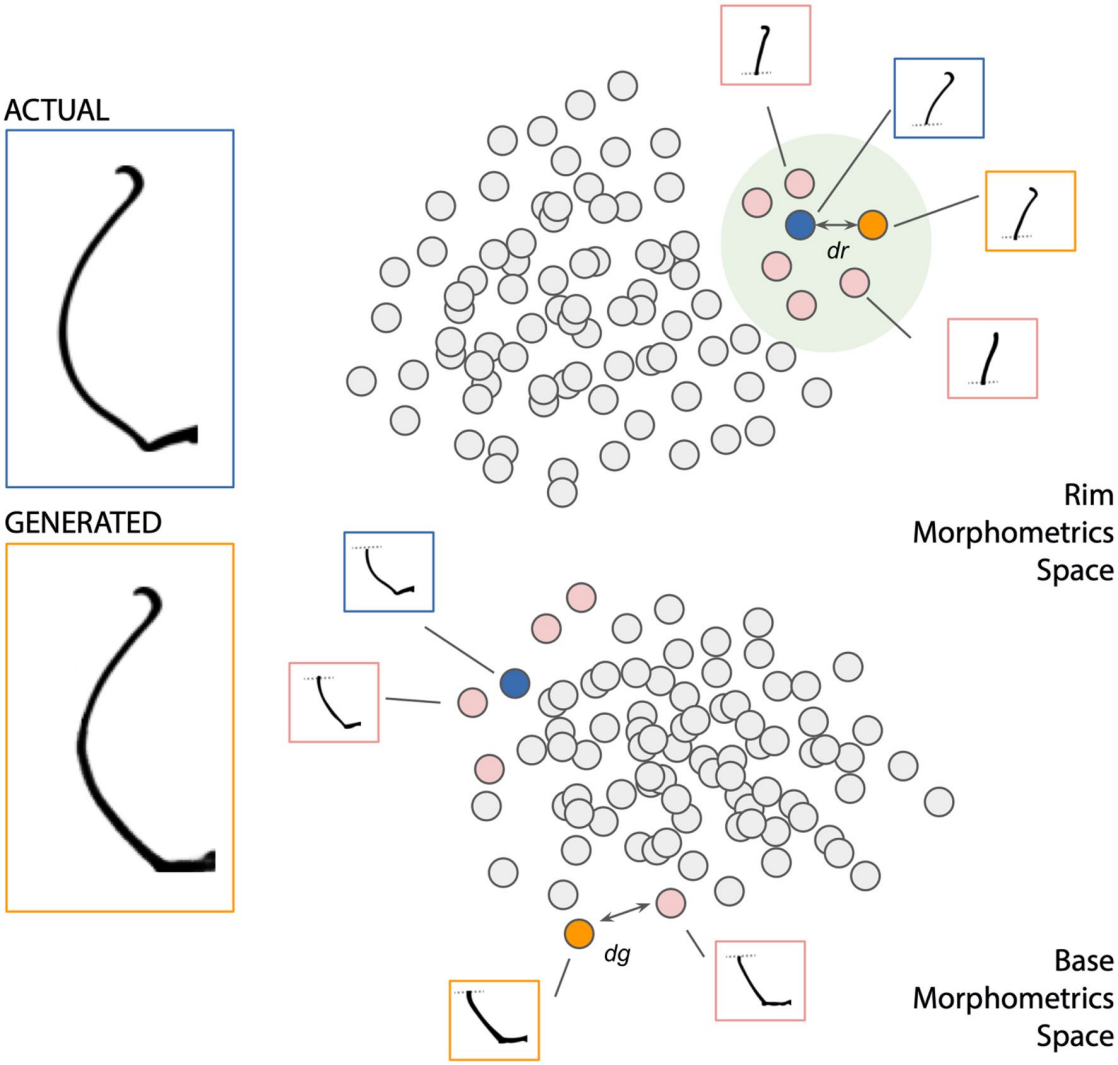
Shape validation. In orange, generated profile with an actual rim. In blue, complete actual Iberian profile. In pink, the k-closest neighbors of the actual fragment (excluding the input rim). *dr* is the distance between the actual and the generated rim. *dg* is the minimum distance (in the base morphometric space) between the generated fragment and its *K* neighbors in the rim morphometric space.

Material evidence of past foraging populations is a prolific research field in archaeology. Among the many factors that inform the Neolithic transition, ceramic potteries are very informative in terms of cultural selection processes. They are one of the most frequently found archaeological artifacts, as well. Since they are usually short-lived, researchers find these artifacts useful to explore chronological and geographical, given that shape and decoration are subject to significant fashion changes over time and space^1^. This gives a basis for dating the archaeological strata, and provides evidence from a large set of valuable data, such as local production, trade relations, and consumer behavior of the local population^2–4^. Several prior studies analyze various aspects of ceramics using complete pottery profiles. Automatic profile classification^5–9^ and feature extraction^10–17^ have been widely studied, ranging from traditional image processing techniques to deep learning approaches. Unfortunately, ceramics are fragile, and therefore most of the actual ceramics recovered from archaeological sites are broken, so the vast majority of the available material appears in fragments. The reassembly of the fragments is a daunting and time-consuming task done almost exclusively by hand, which requires the physical manipulation of the fragments. An intuitive way to understand the fragmentation process, as well as to improve the reconstruction task, is to produce large amounts of potteries imitating the procedures followed by the Iberian craftsmen, breaking them, and then analyzing the resulting sets of fragments. Unfortunately, these and similar manual processing methods for this type of incomplete material are very time-consuming and labor-intensive, even for skilled archaeologists^18^. Due to these factors, there is an increasing interest in automatic pottery reassembly and reconstruction^19–21^ and fragment analysis^22^. Nonetheless, existing work resolves the fragments problem using comparisons between known pieces. The best match within the dataset is the best fragment for that pottery. Here we propose a deep learning approach in which the “best fragment” is artificially generated based on a set of known fragments in the model, thus creating new virtual pottery with the same features as the real ones. The main contributions of this paper are:

- We present IberianGAN, a framework based on generative models that reconstruct pottery profiles from rim or base fragments (see Fig. 1A,B).
- We generate artificial fragment samples using a method to partition the full pottery profiles into two parts (resp. base and rim, see Fig. 1C).
- We evaluate four more approaches for comparison with our architecture. Furthermore, we validate the five methods using a study based on geometric morphometrics (see Fig. 1D and Fig. 2), a domain experts’ validation and open/closed shape classifier (Fig.S1).

### Iberian pottery data

The raw data belong to binary profile images, corresponding to Iberian wheel-made pottery from various archaeological sites of the upper valley of the Guadalquivir River (Spain). The available images consist of a profile view of the pottery, where image resolutions (in pixels), corresponding to size scale, may vary according to the acquisition settings (Fig. S2). We partitioned these images into rim and base portion to simulate the fractures in the profiles. The partitioning criterion and orientation depends on the initial shape (closed or open, see Fig. 1B). The resulting dataset is composed of 1075 images, randomly divided into a training subset containing 752 images (70%), a validation set of 108 (10%), and a test set of 215 images (20% of the total dataset).

### Algorithmic background

GANs have shown remarkable results in various computer vision tasks such as image generation^23,24^, image translation^25,26^, face image synthesis^27–29^ and recently text^30,31^ and audio generation^32^. A typical GAN^33^ framework contains a generative (*G*) and a discriminative (*D*) neural network such that *G* aims to generate realistic samples, while *D* learns to discriminate if a sample is from the real data distribution (*H*_0_) or not. *D*(*x*) should be high when *x* comes from training data and low when *x* comes from the generator. The variable *z* is a latent space vector sampled from a normal distribution. *G*(*z*) represents the generator function which maps the latent vector *z* to data-space of Iberian pottery profiles.

1 $${\text{min}}_{G}\, {\text{max}}_{D} V\left( {D,G} \right) = {\mathbb{E}}_{{x \sim p_{{\text{data }}} \left( x \right)}} \left[ {{\text{log}}D\left( x \right)} \right] + {\mathbb{E}}_{{z \sim p_{z} \left( z \right)}} \left[ {{\text{log}}\left( {1 - D\left( {G\left( x \right)} \right)} \right)} \right]$$

Multiple iterations will inform *G* on how to adjust the generation process to fool *D*. In our case, the data element *x*, corresponds to a binary two-dimensional array containing the pottery profile geometry. *D*(*G*(*z*)) is the probability that the output of the generator *G* is a real sample from the Iberian pottery dataset. *D* tries to maximize (*log D*(*x*)), which is the probability of having a correct classification of actual shapes, while *G* tries to minimize (*log* (1 − *D*(*G*(*x*))), which is the probability of *D* recognizing any of the faked outputs generated by *G*. Deep Convolutional Generative Adversarial Networks (DCGAN)^34^ are among the most popular and successful networks designed for GANs. The model is mainly composed with convolution layers without max pooling or fully connected layers. It uses convolutional stride and transposed convolutions for down-sampling and up-sampling. In other works, the vector *z* is constructed from one or more input images, the generated sample is conditioned by the input. To this type of Autoencoding GAN (AE-GAN) is added a network of encoders that is trained to learn an $E:X \to Z$ function, mapping each real sample to a point (*z*) in latent space^35^. The detailed design and implementation of our proposed generative approach is described in “Materials and methods” section.

## Results

Results from IberianGAN were compared with multiple approaches based on AE-GAN^35^. All approaches contain variations in the architecture or training process (see the “Materials and methods” section). We assess the methods across several generative metrics, a geometric morphometric analysis, a validation based on an open and closed shape classifier, and a validation test made by domain experts. Particularly, to evaluate the quality of images produced by IberianGAN, we computed the following generative metrics: Root Mean Square Error (RMSE), Frechet Inception Distance (FID)^36^, Geometry Score (GS)^37^, and Dice Coefficient^38^. RMSE allows evaluating the generated results in comparison with the actual profiles. RMSE quantifies how different two images are. The smaller an RMSE value, the more similar the profiles are. The metric FID is aimed to compare the distribution of generated images with the distribution of real images. A lower FID value indicates better-quality images, and a higher score indicates a lower quality output. The GS allows comparing the topology of the underlying manifolds for two shapes (in this case, actual pottery and synthetic ones) in a stochastic manner^37^. Low GS values indicate similar topologies between two sets of data. Finally, the Dice coefficient is used to compare two binary images (black or white pixels). The metric takes a normalized value in [0*,* 1], where 0 means two images are completely different, and 1 occurs when both are the same image. In Table 1 we present the performance metrics for the test set from the Iberian dataset. For RMSE, FID, and DC scores, IberianGAN has a significantly better performance when compared with the architectures presented elsewhere.

**Table 1:** Quantitative performance evaluation for different approaches using a dataset test.

Model	RMSE	DICE CONF	FID	GS-SCORE
AE-GAN	0.2354	0.5485	0.0149	0.0194
AE-GAN-MP	0.2312	0.5511	0.0178	0.0147
AE-GAN-MP + MSE	0.2310	0.5653	0.0345	0.0027
AE-GAN-RL	0.2452	0.5010	0.0460	**0.0018**
IberianGAN	**0.1663**	**0.7734**	**0.0045**	0.0337

The best value per metric are in [bold].

**Figure 3:**
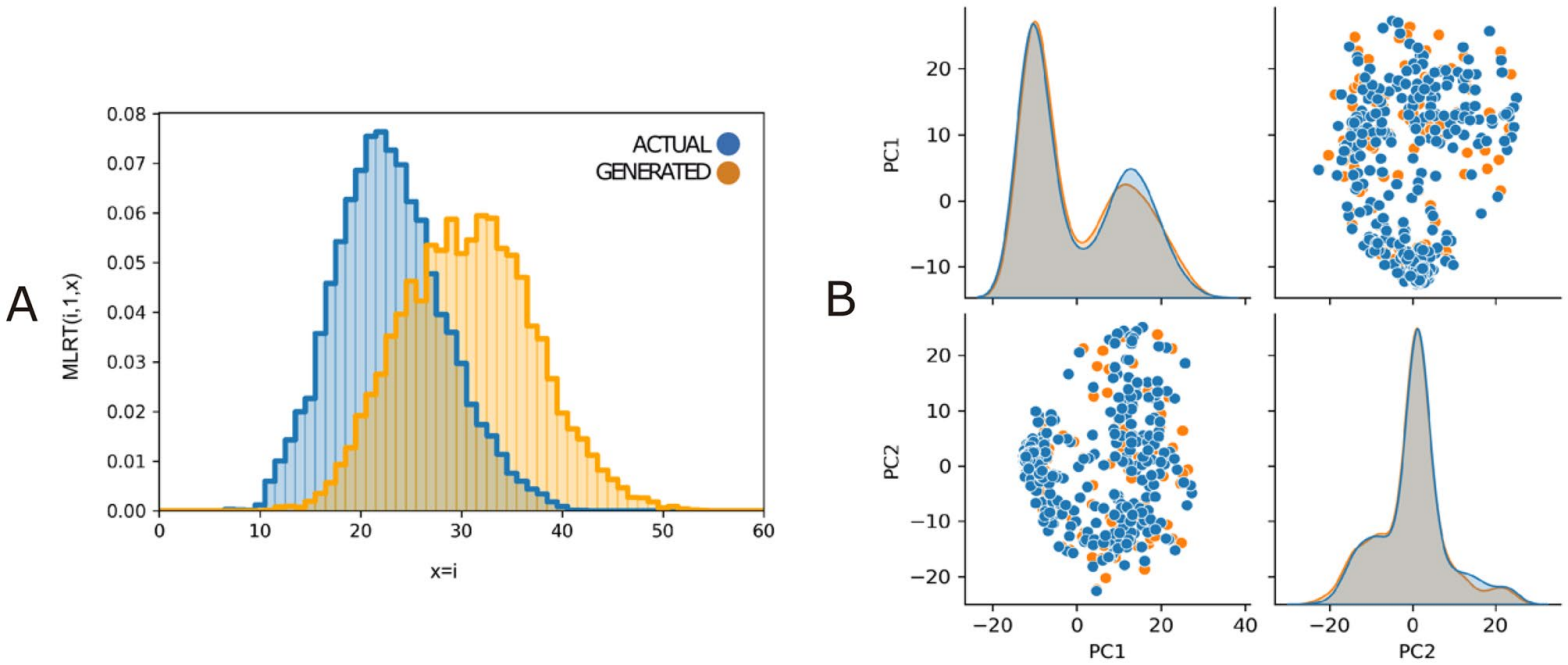
(**A**) GS distribution of the real (blue) and generated (orange) data set. For more information about the GS metric see section “Materials and methods”: Evaluation metrics. (**B**) PCA comparison on the full real dataset and randomly generated 1200 samples.

**Figure 4:**
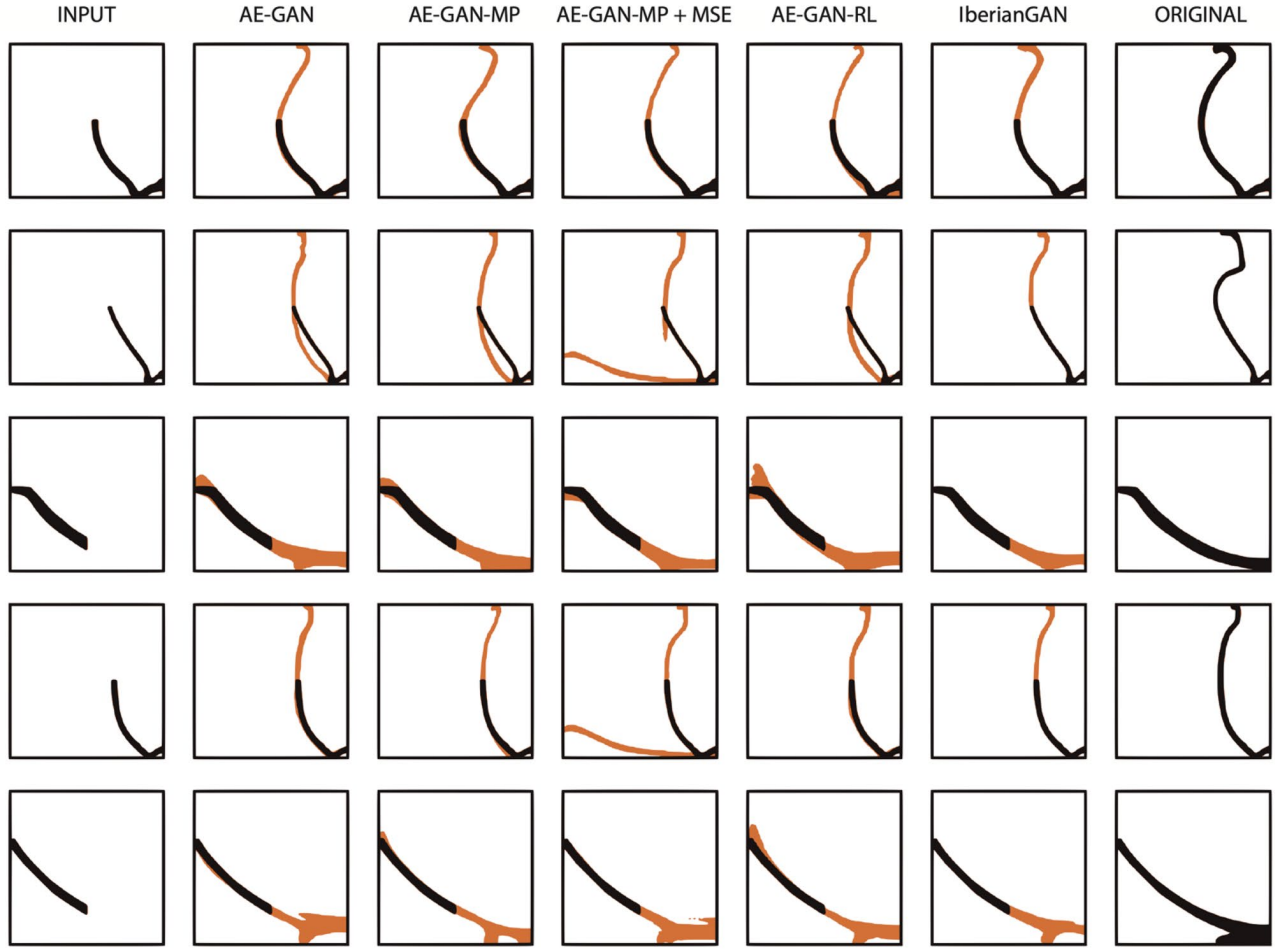
Random examples were sampled to compare the performance of IberianGAN against the other approaches. The generated pottery is in orange. In black is the input fragment.

This means that the generated profiles have a similar geometric distribution with respect to the actual samples, and thus the resulting potteries are comparable to the actual samples. A proposed alternative with reinforcement learning (AE-GAN-LR) improves the topology similarity (GS metric). Nonetheless, the topological similarity is not the most relevant factor, and that there is indeed an overlap between synthetic topologies generated by AE-GAN-LR and IberianGAN (see Fig. 3A) we consider that the synthetic samples generated by the latter can be regarded as topologically correct as compared to the actual samples. Furthermore, we evaluated the distribution of data qualitatively. For this, we created a feature space using Principal Component Analysis (PCA) with the images from actual and generated pottery. In Fig. 3B, we observe that the distribution in this feature space of the actual images is similar to the distribution of images generated with IberianGAN. We qualitatively compare the results of all approaches. In Fig. 4, we show some results using the same input and comparing it with the original image of the dataset. As observed, IberianGAN looks at the input image and completes the fragment with convincing results (see further results in Fig. S4). Given the results mentioned above, IberianGAN can be used satisfactorily to estimate missing fragments and provide realistic, complete pottery profiles, maintaining the geometric properties of original potteries.

### Shape validation

In the real profile dataset, the base shape of a profile appears in combination with only a subset of the entire rims set (and vice-versa), i.e. not all base/rim combinations are present in real profiles. This is because the entire structure of the pottery is usually designed to serve only one purpose (e.g. liquid storage, cooking, transportation, drinking, ritual, etc.). Some base/rim combinations would create useless or impractical pots (e.g., with a very small base and a large rim). A similar effect is seen when analyzing the design of the projectile point^39^ where the variations of the designs of the stem and the blade (two parts of a projectile point) of these artifacts are studied in a modular way to determine the relation in the designs of its shapes. Thus, we evaluate the ability of IberianGAN to generate rims with a valid shape from existing bases and vice-versa. Based on^39^, we extracted semi-landmarks to analyze the shape of the generated fragments. Using the profile dataset of actual pottery, we created a morphometric space using the semi- landmarks of the fragments as input for a PCA. We worked with four morphometric spaces, two for closed and two for open pottery shapes, each one containing its corresponding rims and bases. In order to obtain a metric that allows us to compare generated profiles, we analyze the Euclidean distance between the generated fragments and the real pottery profiles in these morphometric spaces (see a graphical description in Fig. 2). Given a pot generated from an existing fragment (e.g., a rim), we first divide the generated profile and locate the two resulting halves on their corresponding spaces, and then analyze the distance between the actual and the generated fragments (*dr* in Fig. 2). To evaluate the other half of the generated profile, we use the closest *K* fragments (*K* = 50) to the real one (the input fragment) in the first space, and we place its pairs in the other space (in our example, the space generated for all of the real bases). We calculate the minimum distance in this space between the generated fragment and its neighbors in the first space (*dg* in Fig. 2). This type of morphometric validation establishes the ability of the method to generate a fragment with an actual shape from an input fragment. In Table 2 we show the mean Euclidean distances in all the approaches tested in this work (see “Materials and methods” section). The table presents two parts, corresponding to open and closed shapes. We considered two scenarios, when the input is a rim or is a base. As IberianGAN only generated the unknown fragment, the distances between the input and the known fragment are close to zero. In the approaches where the network generated the shape for the entire profile, the distances between known and unknown fragments are similar.

**Table 2:** Euclidean distances in morphometric spaces for open and closed Iberian pottery shapes.

Model	Known rim (*dr*)	Generated base (*dg*)	Known base (*dr*)	Generated rim (*dg*)
**Closed shapes**				
AE-GAN	0.0620 ± 0.0288	0.0191 ± 0.0083	0.1407 ± 0.0859	0.0219 ± 0.0090
AE-GAN-MP	0.0900 ± 0.0398	0.0250 ± 0.0141	0.1197 ± 0.0771	0.0289 ± 0.0101
AE-GAN-MP+MSE	0.0756 ± 0.0263	0.0218 ± 0.0138	0.1701 ± 0.1183	**0.0213** ± **0.0123**
AE-GAN-RL	0.0813 ± 0.0284	**0.0181** ± **0.0094**	0.1507 ± 0.1024	0.0235 ± 0.0126
IberianGAN	**0.0006** ± **0.0023**	0.0206 ± 0.0110	**0.0001** ± **0.0009**	0.0265 ± 0.0088
**Open shapes**				
AE-GAN	0.0356 ± 0.0261	**0.0360** ± **0.0184**	0.1728 ± 0.0907	0.0127 ± 0.0069
AE-GAN-MP	0.0337 ± 0.0275	0.0364 ± 0.0204	0.1670 ± 0.1176	0.0147 ± 0.0096
AE-GAN-MP + MSE	0.0222 ± 0.0186	0.0363 ± 0.0200	0.2080 ± 0.1317	**0.0126** ± **0.0072**
AE-GAN-LR	0.0472 ± 0.0346	0.0474 ± 0.0285	0.1536 ± 0.1111	0.0140 ± 0.0079
IberianGAN	**0.0005** ± **0.001**	0.0418 ± 0.0286	**0.0002** ± **0.0008**	0.0204 ± 0.0215

The best value per metric are in [bold].

### Sample quality validation based on open and closed shape classifier

Separately from the generative modes, we trained a binary classifier. This model is capable of classifying open and closed vessel profiles. We used pre-trained weights of ResNet-18^40^. This validation aims to verify that the data generated by the different models is able to imitate the real samples and that the classifier can predict the correct classes even when trained with only real data samples. Table S1 shows the classification metrics using the different datasets. In particular, it can be seen that the classifier is not affected by the generated data. Notably, the metrics improve compared to the actual test data portion in all cases. Additionally, in Fig. S1, we can see a graphic representation of the sensitivity versus the specificity of the classifier as the discrimination threshold is varied. This type of result shows that the generated new samples are similar in their distribution and shape to the real data. In addition, note that they do not affect the accuracy of the classifier.

### Domain expert validation

We designed an experiment for domain archaeology experts to evaluate the capability of IberianGAN to create pottery profiles with an adequate Iberian style. For this purpose, we present in the form of an online questionnaire a set of images (see Fig. S3) to six archaeologists specialized in Iberian culture. In the survey, we display a random selection of 20 images where half of them correspond to actual Iberian pottery profiles and half IberianGAN-generated. Each image has a multiple-choice to rate it between 0 and 5 to determine the level of similarity with an Iberian style, where 0 means unrelated to the Iberian Style, and 5 means fully within Iberian Style. Overall, generated samples rated 3*.*88 on average with a standard deviation in 1*.*43 across all archaeologists and actual samples rated 3*.*93 ± 1*.*45. To conclude, the archaeologists consider that the potteries generated have on average an Iberian style similar to that found in actual potteries. This is important since IberianGAN is capable of generating an Iberian-style pottery from an incomplete fragment.

## Discussion

Ceramics are one of the most frequently found archaeological artifacts and constitute the central remains usually used to investigate variations in style, materials employed, and manufacturing techniques. Exploring diachronic and geographical variation in pottery is of key importance to reconstruct the dynamics of the Neolithic transition in different regions. However, ceramics are fragile, and therefore most of the recovered material from archaeological sites is broken. Consequently, the available samples appear in fragments. The reassembly of the fragments is a daunting and time-consuming task made almost exclusively by hand, which requires the physical manipulation of the ceramic shreds. Thus, a generative approach, such as IberianGAN, that automatically processes fragments and provides a reconstruction analysis can assist archaeologists in the reassembly process.

Such an approach has a broader impact by providing a general framework for object reassembly. Our proposed framework is flexible to work on different ceramic datasets that present a variety of fractured materials (see results from Roman Pottery in Fig. S6). IberianGAN could be used beyond just ceramic pottery in order to reconstruct other archaeological (e.g. projectile points, historical buildings, etc.) and anthropological remains (e.g. crania, postcranial bones, etc).

We have evaluated the performance of IberianGAN on the basis of three different but complementary approaches: (a) classical metrics to evaluate the generative process of images (see Table 1 and Fig 2); (b) shape analysis based on pottery structure (see section “Results”: Shape validation), and (c) validation via independent examination of archaeologists specialized in Iberian heritage (“Results”: Domain expert validation).

Results obtained under the three approaches suggest that our approach is capable of generating potteries that satisfy the image, pottery morphometric structure, and expert validation criteria. While encouraging performance is achieved with IberianGAN for the prediction of fragments in the database of Iberian wheel-made pottery, some limitations need to be addressed. In general, archaeologists work with fragments belonging to the base or top of the pottery. Therefore the network was trained always using a base or rim fragment, meaning that the model will always position a fragment as a base or rim. Furthermore, our approach uses large fragments during training and evaluation. Additional studies are needed to determine the minimum accepted size of a fragment for the model to perform as expected. Nonetheless, we believe our proposed framework is the first step towards broader use of generative networks for the recognition and assembly of fragments, which will open new avenues of research related to applications on different measurements of fragments and even in 3D ceramics in particular and objects in general.

## Related work

Previous research on pottery includes both classical approaches, based on the comparative analysis of shape, dimensions, decoration, technological elements, color, geometric characteristics, axis of symmetry, used materials, etc; and novel methods based on machine learning techniques in general and deep learning in particular applied towards ceramic characterization. As a whole, pottery profiles were used in the context of classification^5–9^, and to study variations in shape and/or style attributes^10–17^. As previously stated, not all the potteries found in the excavations are complete; that is why it is critical to improve characterization methods aimed to identify fragmented ceramics. Rasheed et al^19^ presented a method based on a polynomial function for reconstructing pottery from archaeological fragments. Given an image of a fragment, the edge curve is extracted and approximated by a polynomial function to obtain a coefficient vector. The best matching between pairwise pottery fragments is done according to the relationship of their coefficients.

Other authors proposed a method to generate missing pieces of an archaeological find^20,21^ departing from a 3D model. In the area where the missing fragments are supposed to be, sketches are created through reverse modeling and consequently used to design the missing fragments. Finally, the digital reproduction of the missing part is achieved Additive Manufacturing technology.

GANs have shown remarkable results in various application domains. Their ability to learn complex distributions and generate semantically meaningful samples has led to multiple variations in network design and novel training techniques (GANs^33^, conditional GANs^41^, InfoGAN^42^, BAGAN^43^). Customized loss functions (Content loss^44^, Cycle consistency loss^45^), and domain adaptation approaches (ADDA^46^, CycleGAN^47^), etc. A more comprehensive review of the different GAN variants and training techniques can be found in^48–50^.

Furthermore, there are multiple examples of GANs applied to cultural heritage domains. For instance, techniques such as automated image style transfer^51^, were used to develop a model aimed to generate Cantonese porcelain styled images departing from user-defined masks. Similar techniques were also applied to material degradation^47,52–56^. Hermoza et al.^57^, for instance, introduced ORGAN, a 3D reconstruction GAN to restore archaeological objects. This is based on an encoder-decoder 3D DNN on top of a GAN based on cGANs^41^. This network can predict the missing parts of an incomplete object. A similar approach is followed in ^58^, where a Z-GAN translates a single image of a damaged object into voxels in order to reconstruct the original piece. Both studies address the problem of the prediction of missing geometry on damaged objects that have been 3D modeled and voxelized. More specifically, these studies depart from the assumption that man-made objects exhibit some kind of structure and regularity. The most common type of structure used is symmetry. Starting from a GAN they learn the structure and regularity of a collection of known objects and use it to complete and repair incomplete damaged objects. Another example of cultural heritage preservation can be found in reference^59^, where an image completion approach is adapted^60^ for the curation and completion of damaged artwork.

## Materials and methods

We designed, trained, and evaluated five different generative networks based on AE-GAN but used multiple training procedures during the experimentation phase. In this section, we detailed each incremental strategy applied in the process and their corresponding hyperparameters and training techniques and setup. The data and source code with the hyperparameter setup and different approaches analyzed in this study are openly available in IberianGAN at https://github.com/ celiacintas/vasijas/tree/iberianGAN for extension and replication purposes.

### Experimental setup and training

All the resulting networks were trained for 5000 epochs using Binary Cross Entropy as a loss function, at a learning rate 2 × 10^−4^ for the generative network (G) and 2 × 10^−5^ for the discriminator (*D*). To optimize the training process of all models, we scaled the images to a uniform resolution of 128 × 128 pixels and inverted the colors. We applied data augmentation, particularly a random rotation (between 0 and 45 degrees). We used ADAM optimization^61^ for both *G* and *D* with *β*_1_ = 0*.*5 and *β*_2_ = 0*.*999 and used Binary Cross Entropy as the loss function. Particularly, for the training of *D*, we used Label Smoothing^62^, the real set is represented with a random number between 0*.*7 and 1*.*2 and the generated set with 0*.*0 and 0*.*33.

### AE-GAN incremental variations and IberianGAN

Initially, we trained a typical AE-GAN to generate a complete pottery profile. We implemented a generator (*G*) with an architecture that allows two images as input and a discriminator (*D*) with three input images (the two inputs and the generated image). During training and to speed the convergence process of G, we create different input types with the same probability and select a pair of images. The possible input types were rim/base (or base/rim), base/black image or rim/black image (see Fig. S5-A). Subsequently, aiming to obtain a translation from the input fragment to the complete pottery profile, we modify the architecture of the encoder in the AE-GAN part of the generator, called AE-GAN-MP. In this case, the generator encoder processes one input image at a time. We do this to embed the input images separately and apply a max-pooling layer to join the two representations, see Fig. S5-B. This modification allows more variability in the representation for generating the full profile.

Additionally, we define a new loss function for training the generator of the AE-GAN-MP architecture. Using the strategy of multiple types of input (rim/base, base/rim, rim/black, and base/black), we compute this new loss function only when the inputs are complete (e.g., rim/base or base/rim). For this, we use Mean Square Error (MSELoss) defined as follows:

2 $${\text{L}}\left( {y,\hat{y}} \right) = \frac{1}{N}\sum_{i = 0}^{N} \left( {y - \hat{y}_{i} } \right)^{2}$$

where $\hat{y}$ is the predicted pottery and ${\text{y}}$ is the real example. The goal is that the generator minimizes the MSE error between the result and the target (real pottery profile). Finally, to obtain a stronger relationship between the inputs and the generated pottery we design a strategy to modify the resulting pottery (or iterate to get a more precise result). We do this by using the input with the previous result to generate new pottery (the final result) with two iterations. The intermediate result adds to the input using image matrix operations, see Fig. S5-C. We called this approach AE-GAN with reinforcement learning (AE-GAN-RL).

IberianGAN is based on the AE-GAN, where the generator is an Autoencoding network ${\text{Encode}}\left( {\text{x}} \right) \to {\text{z }} \in {\text{R}}^{{\text{m}}} ,{\text{ Decode}}\left( {\text{Z}} \right) \to {\text{x}}^{{\prime }}$, where ${\text{x}} \in \left[ {0,1} \right]^{{{\text{m}} \times {\text{m}}}}$, is the input fragment, a binary two-dimensional array containing the fragment shape information, and x' is a missing generated part. To train the discriminator network, we use D(y) where ${\text{y }} = {\text{ x }} + {\text{ x}}^{{\prime }}$ for the examples generated. At this point, the network generates only an unknown fragment and the discriminator is trained with the complete profile. As IberianGAN only generates the missing fragment, for its training process, it is not necessary to use two images as input (see Fig. 1A). For training, we only use an image that corresponds to the base or the rim of the profile. The complete definition, implementation, training and evaluation of IberianGAN can be found here: https://github.com/celiacintas/vasijas/tree/iberianGAN.

### Evaluation metrics

In this section, we show the process of evaluating the quality of the generated samples. To compare the results of the different approaches, we use two approaches. First, a set of measurements used to evaluate GANs, these metrics refer to the general distribution of all the potteries generated. Additionally, we use metrics comparing the result obtained with the actual potteries, for example, to evaluate the known fragments in the potteries generated. For the first type, we consider evaluating the distribution and shape of the generated profiles. First, we use Frechet Inception Distance (FID)^36^, which is currently one of the most common metrics to evaluate GANs^63^. FID allows the quantification of the differences in the density of two distributions in the high-dimensional feature space of an InceptionV3 classifier^64^. In detail, FID embeds the images in a descriptor space (defined by an intermediate layer of Inception-V3) with a high level of abstraction. This feature space is used to calculate the mean and variance of the generated data and the actual data. The Fletcher distance is calculated between these distributions. FID is calculate following this equation:

3 $$FID\left( {r,g} \right) = ||\mu_{r} - \mu_{g}||^{2}_{2} + {\text{Tr}}\left( {\Sigma_{r} + \Sigma_{g} - 2\sqrt {\Sigma_{r} \Sigma_{g} } } \right)$$

where (*µ*_*r*_, Σ_*r*_) and (*µ*_*g*_, Σ_*g*_) are the mean and co-variance of the actual data and the generated distributions, respectively. Small distances indicate that the distribution of the data is similar, in our case, that the generated potteries have a distribution similar to the real ones. FID is based on a classifier network. It has been shown that this type of metric focuses on textures rather than shapes^65^, so we decided to evaluate the approaches with a shape-based metric, Geometry Score (GS)^37^.

GS is a metric for comparing the topological properties of two data sets. Formally GS is the l2 distance between means of the relative lifetime vectors (RLT) associated with the two groups of images. The RLT of a group of images (encoded in a feature space, for example) is an infinite vector (*v*_1_*, v*_2_*, ..., v*_*i*_) where the *i-th* entry is a measure of persistent intervals that have a persistent homologous group rank equal to *i*. *v*_*i*_ is defined as follows:

4 $$v_{i} = \frac{1}{{d_{{n\left( {n - 1} \right)/2}} }}\sum_{j = 1}^{{n\left( {n - 1} \right)/2}} I_{j} \left( {d_{j} + 1 - d_{j} } \right)$$

where *I*_*j*_ = 1 is the rank of a persistent homologous group with dimension 1 in the interval [*d *_*j*_*, d *_*j*_ + 1] is *i* and *I*_*j*_ = 0 is the opposite^37^. Low GS values indicate similar topology between the set of images. On the other hand, for the second group of metrics, we evaluate the results against the complete potteries. It is important to clarify that we do not try that the results are the same as the actual pottery since it is generated only with a fragment, with this objective, we use two frequent metrics in image processing, Root Mean Square Error (RMSE) and DICE Coefficient^38^.

RMSE is a metric that enables similarity comparisons between two samples (pottery profiles in this case). This is measured using the square root of the average of the squared differences between the pixels in the generated image and the actual image. The RMSE between a actual profile image, (image *d*) and the generated image, (image *f* ) is given by

5 $$\sqrt {\frac{1}{n}\sum_{i = 1}^{n} \left( {d_{i} - f_{i} } \right)^{2} }$$

This metric is calculated pixel by pixel, where *d*_*i*_ and *f*_*i*_ are the pixels of the image D and F respectively. In this formula, low RMSE values show a minor error. The DICE coefficient allows to evaluate the geometry between the generated profile and the real one. This metric is commonly used to evaluate results in segmentation networks^66^. That is why to calculate the DICE coefficient, the images must be binary (black and white). This coefficient evaluates the images as two overlays of shapes. To do this, the region of the generated image and the region of the actual profile are calculated. Given the generated profile A and the real profile B, DICE is calculated^38^:

6 $$DICE = \frac{{2\left| {A \cup B} \right|}}{\left| A \right| + \left| B \right|}$$

where *A* and *B* is the size in pixel of the profile. The maximum value of the metric is 1 when the shape is identical to the real one and 0 when the total shape does not match.

## Supplementary Information

Supplementary Information.

## Data Availability

The data and code that support the findings of this study are openly available in IberianGAN at https://github.com/celiacintas/vasijas/tree/iberianGAN for extension and replication purposes.
