# Supplementary material for: Reconstruction of Iberian ceramic potteries using generative adversarial networks
Source: Sci Rep. 2022 Jun 23;12:10644. doi: 10.1038/s41598-022-14910-7 (PMC9225991; doi:10.1038/s41598-022-14910-7)
Supplement: Supplementary file 1 — Supplementary Information. [file 41598_2022_14910_MOESM1_ESM.pdf]

Supplementary Information

Table S1. Performance metrics for open-close shapes classifier from test data.

Dataset	Precision	Recall	F1-Score	AUC
Test (Original Dataset)	0.90	0.89	0.88	0.998
Generated for AE-GAN	0.93	0.92	0.92	0.991
Generated for AE-GAN-MP	0.93	0.93	0.93	0.994
Generated for AE-GAN-MP + MSE	0.91	0.90	0.90	0.994
Generated for AE-GAN-RL	0.94	0.94	0.93	0.994
Generated for IberianGAN	0.90	0.90	0.89	0.998

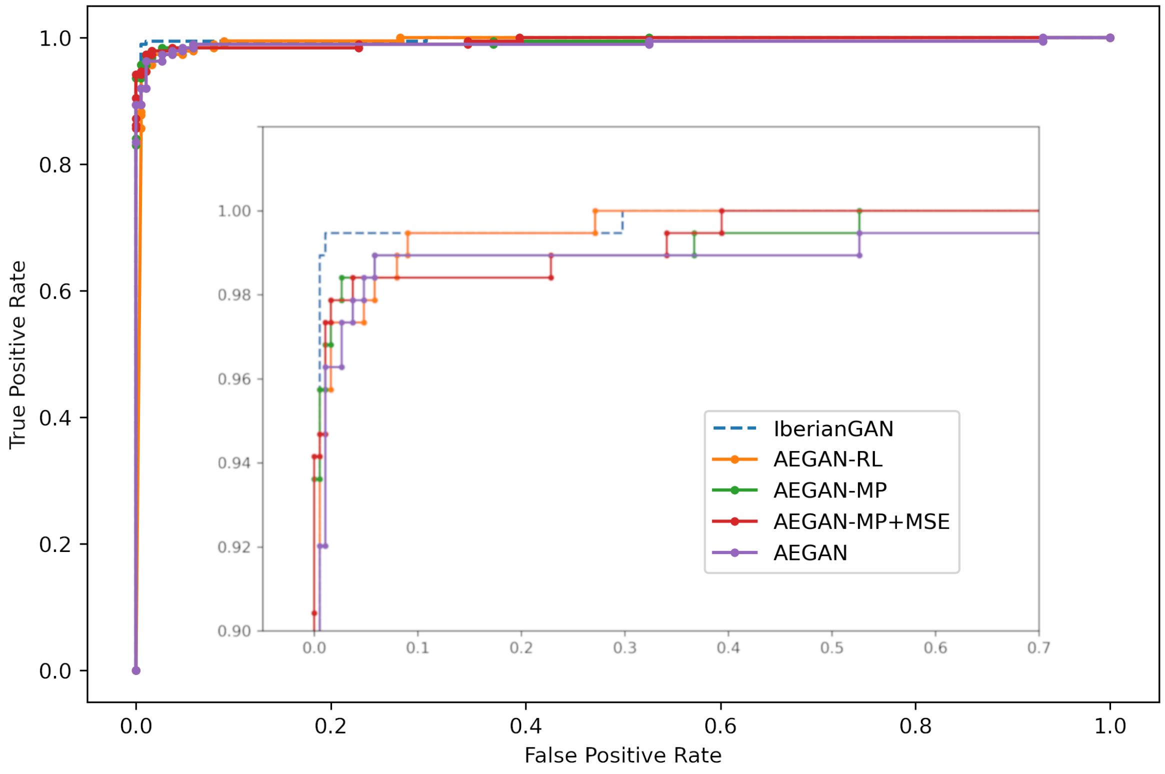

Figure S1. Receiver Operator Characteristic (ROC) curves using different generative approaches. The Area Under the ROC Curve of each method is IberianGAN: $AUCROC = 0.998$, AEGAN-RL, AEGAN-MP, AEGAN-MP+MSE: $AUCROC = 0.994$ and AEGAN: $AUCROC = 0.991$.

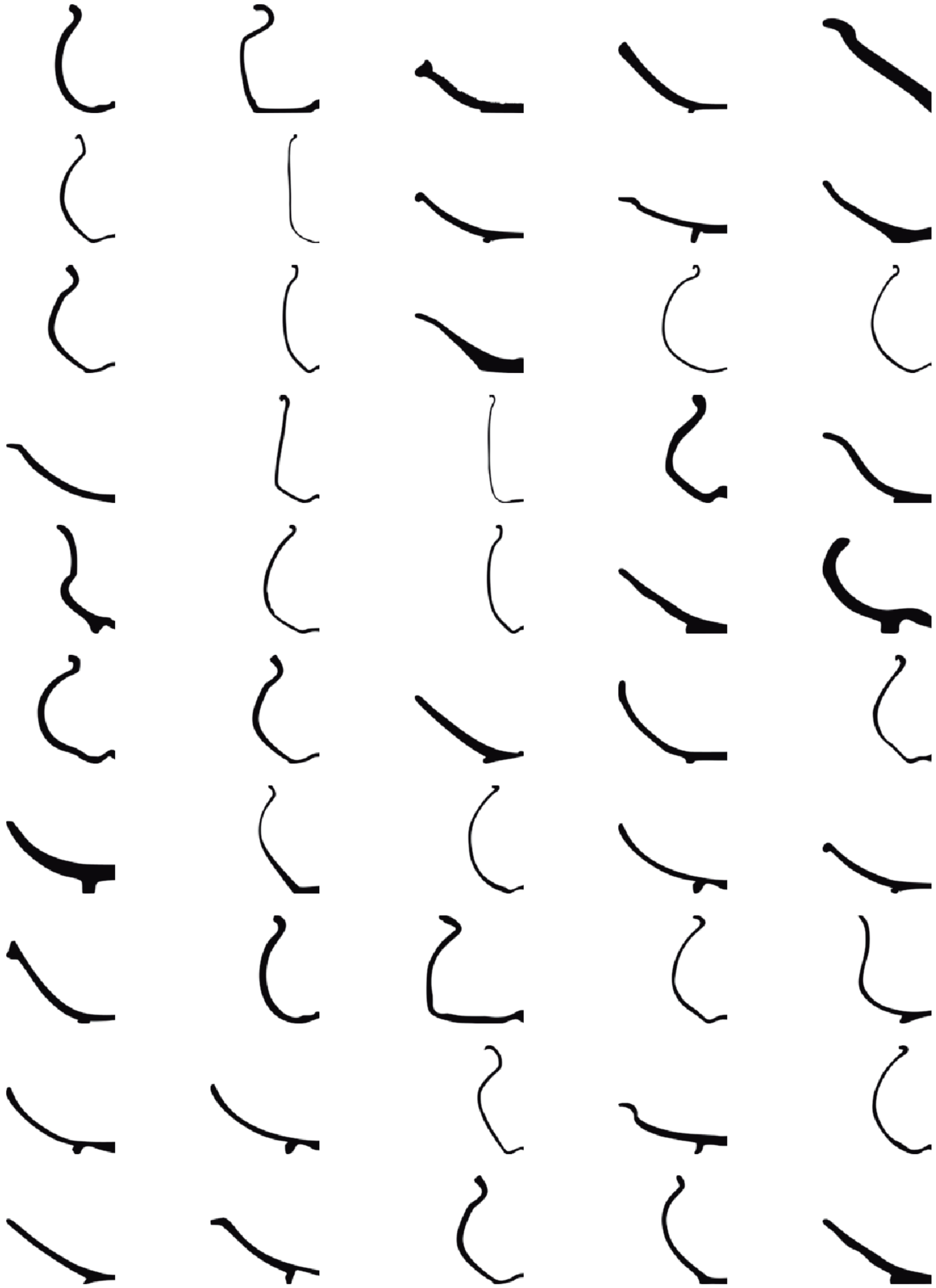

Figure S2. Example of profile images dataset corresponding to Iberian wheel-made pottery from various archaeological sites of the upper valley of the Guadalquivir River (Spain).

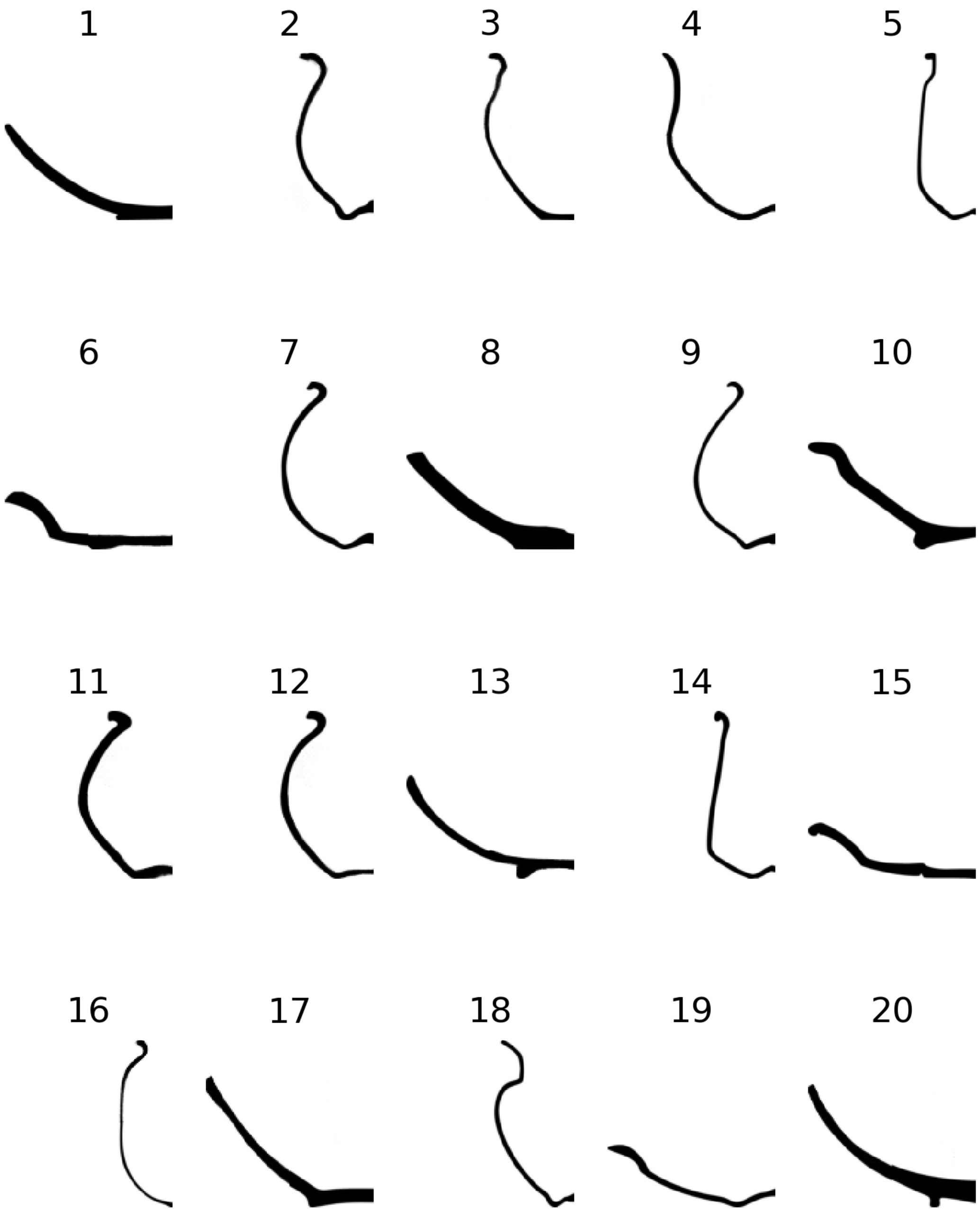

Figure S3. Twenty Iberian pottery profiles examples used in the domain expert evaluation survey, 10 actual samples (indexes: 1,5,7,8,9,10,14,15,18,19) and 10 generated samples (indexes: 2,3,4,6,11,12,13,16,17,20). Each image has a multiple-choice to rate it between 0 and 5 to determine the level of similarity with an Iberian style (0 is means unrelated to Iberian Style, and 5 is means fully within Iberian Style).

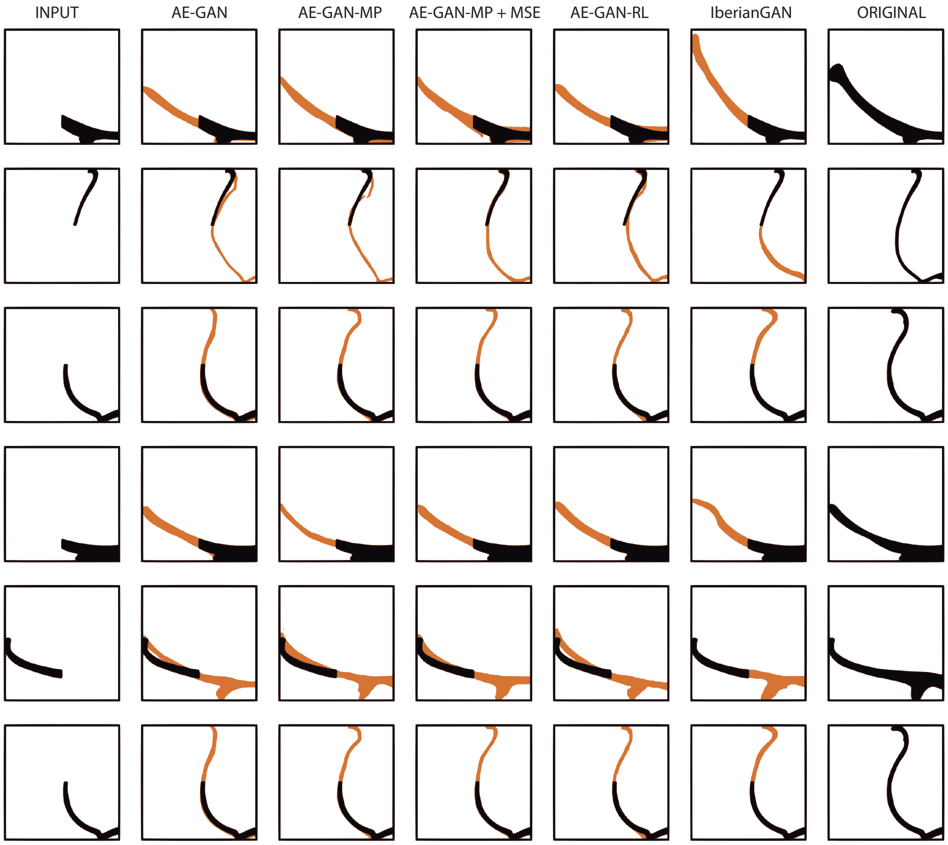

Figure S4. Random examples were sampled to compare the performance of IberianGAN against the other approaches, all of them with 5000 epochs. The generated pottery is in orange and the input fragment is in black.

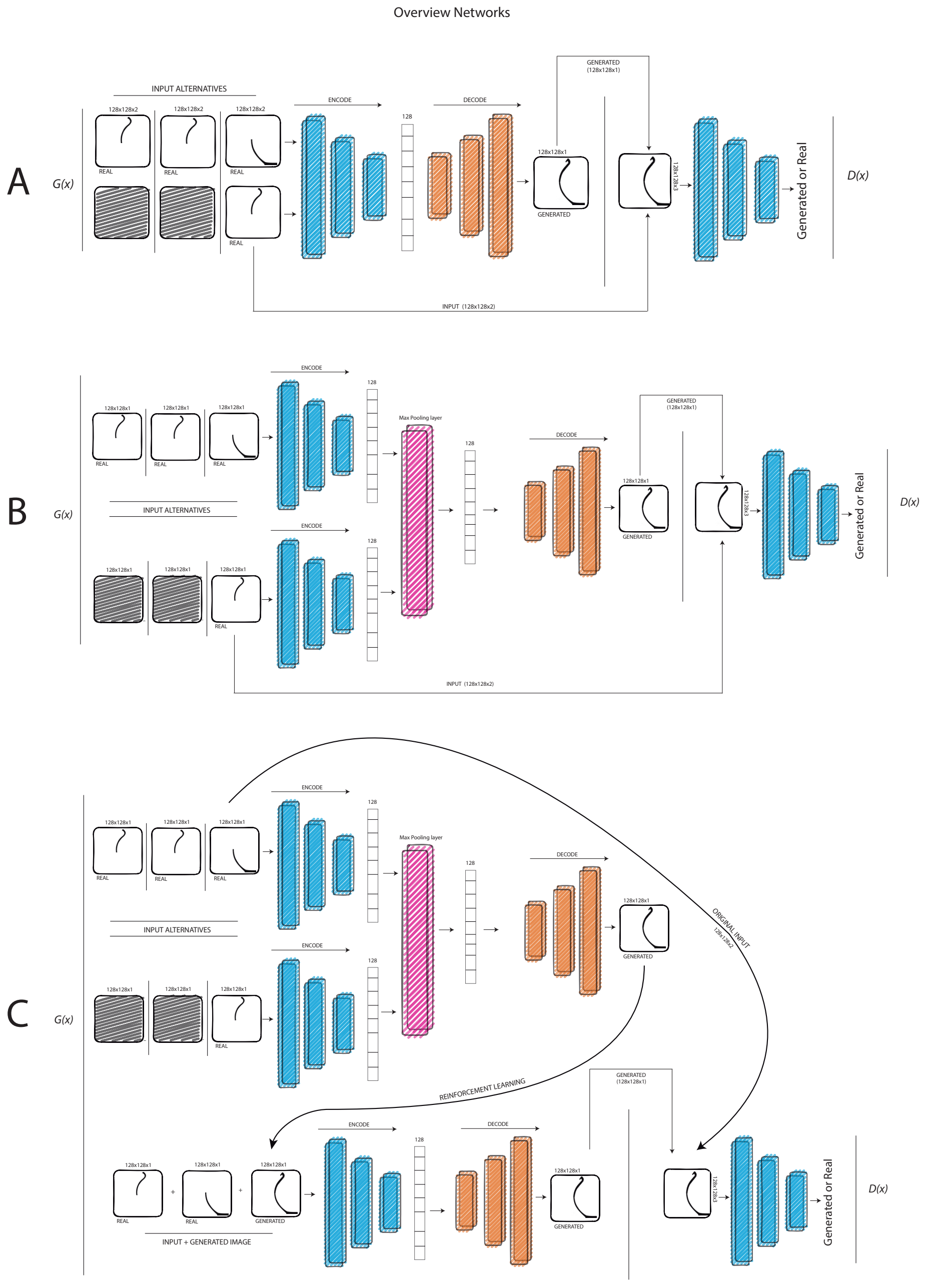

Figure S5. Networks architectures and approaches based on AE-GAN. A, B, C show the strategy of train, changes in the architecture and design of AE-GAN, AE-GAN-MP and AE-GAN-RL respectively. .

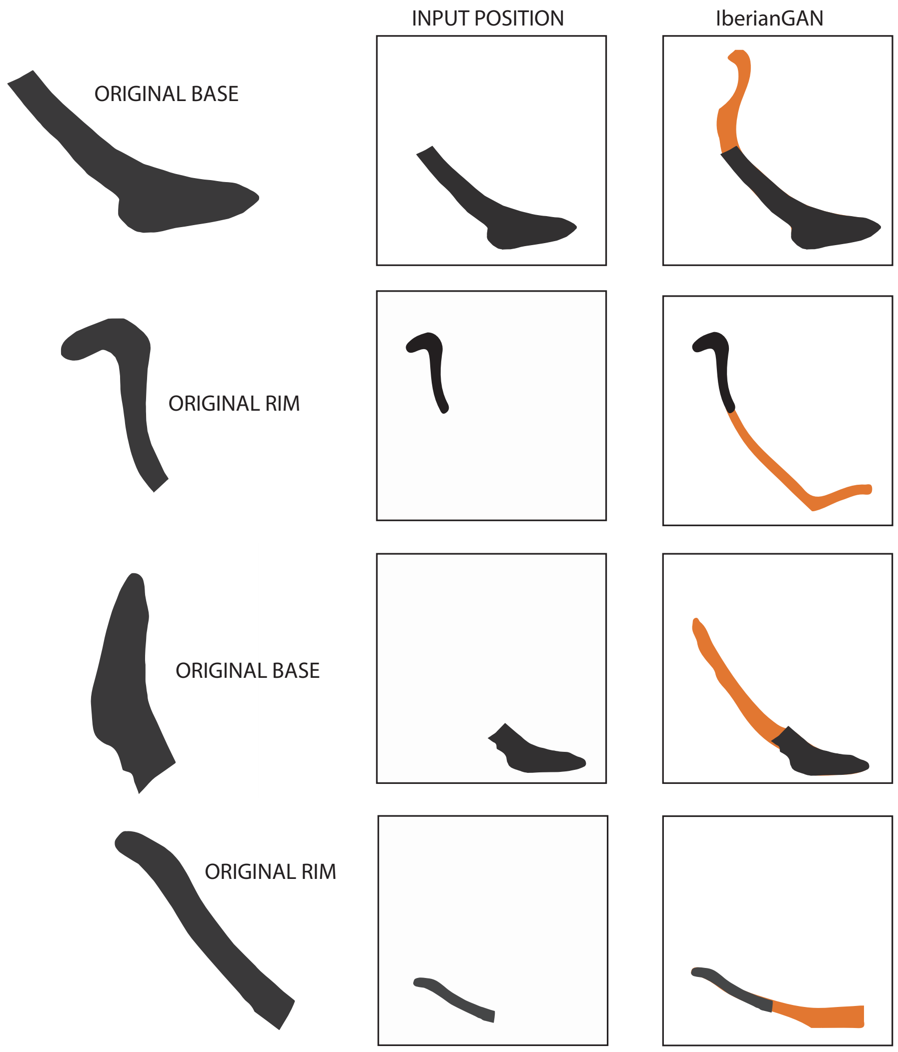

Figure S6. Profile of actual fragments of Roman pottery found at the ancient city of Cástulo, Andalusian province of Jaén, in south-central Spain. The figure shows the performance of IberianGAN with actual fragments of the Roman dataset.
